# Fascicle differentiation of upper extremity nerves on high-resolution ultrasound with multimodal microscopic verification

**DOI:** 10.1038/s41598-024-84396-y

**Published:** 2025-01-02

**Authors:** Luka Pušnik, Barbora Radochová, Jiří Janáček, František Saudek, Igor Serša, Erika Cvetko, Nejc Umek, Žiga Snoj

**Affiliations:** 1 Institute of Anatomy, Faculty of Medicine, University of Ljubljana, Korytkova 2, 1000 Ljubljana, Slovenia; 2 Laboratory of Biomathematics, Institute of Physiology, The Czech Academy of Sciences, Prague, Czech Republic; 3 Diabetes Centre, Institute for Clinical and Experimental Medicine, Prague, Czech Republic; 4 Department of Condensed Matter Physics, Jožef Stefan Institute, Ljubljana, Slovenia; 5 Department of Radiology, Faculty of Medicine, University of Ljubljana, Ljubljana, Slovenia; 6 Institute of Radiology, University Medical Centre Ljubljana, Ljubljana, Slovenia

**Keywords:** Fascicle differentiation, MR neurography, Nerve anatomy, Peripheral nerve imaging, Ultrasonography, Preclinical research, Translational research, Peripheral nervous system

## Abstract

This study aimed to compare the fascicular anatomy of upper limb nerves visualized using in situ high-resolution ultrasound (HRUS) with ex vivo imaging modalities, namely, magnetic resonance microscopy (MRM), histological cross-sections (HCS), and optical projection tomography (OPT). The median, ulnar, and superficial branch of radial nerve (*n* = 41) were visualized in 14 cadaveric upper limbs using 22-MHz HRUS. Subsequently, the nerves were excised, imaged with different microscopic techniques, and their morphometric properties were compared. HRUS accurately differentiated 51–74% of fascicles, while MRM detected 87–92% of fascicles when compared to the referential HCS. Among the compared modalities, HRUS demonstrated the smallest fascicular ratios and fascicular cross-sectional areas, but the largest nerve cross-sectional areas. The probability of a fascicle depicted on HRUS representing a cluster of multiple fascicles on the referential HCS increased with the fascicular size, with some differences observed between the larger median and ulnar nerves and the smaller radial nerves. Accordingly, HRUS fascicle differentiation necessitates cautious interpretation, as larger fascicles are more likely to represent clusters. Although HCS is considered the reference modality, alterations in nerve cross-sectional areas or roundness during sample processing should be acknowledged.

## Introduction

Peripheral nerves consist of nerve fibers grouped into fascicles, which are enclosed by a thin layer of perineurium, and embedded in loose interfascicular epineurium^1^. The nerve microanatomy is complex, and several approaches have been used to study it. The initial studies pioneered by Sunderland and colleagues relied predominantly on analysis of nerve histological cross-sections (HCS), where they observed rapid transitions in fascicular anatomy^2,3^. More recent research has characterized fascicular organization using sophisticated immunohistochemical techniques^4^. Advanced imaging techniques such as micro-computed tomography, optical projection tomography (OPT), and magnetic resonance microscopy (MRM) have enabled more precise depiction of fascicular interconnections which are partly responsible for changes in fascicular anatomy, highlighting the necessity for clinical translation^5–7^.

The depiction of nerve fascicles in vivo remains difficult, and a thorough evaluation of available imaging methods is needed to understand and define their potential or capabilities to visualize nerve fascicular anatomy^8,9^. Recent advancements in hardware and software for high-resolution ultrasound imaging (HRUS) have enhanced image quality, facilitating the delineation of finer anatomical details of peripheral nerves^10^. Accordingly, HRUS has become an indispensable adjunct in non-invasive, real-time imaging of nerves in clinical practice, particularly due to its cost-effectiveness and excellent spatial resolution^11^. Visualizing nerve fascicles can aid in identifying their involvement in traumatic nerve lesions, thereby informing clinical decision-making and determining the necessity of surgical intervention^12^. Additionally, the depiction of individual nerve fascicles on HRUS is particularly important, given that some neuropathies may differentially affect individual fascicles^13^.

There is currently no consensus in terminology on reporting intraneural structure using radiologic modalities. Some authors describe a ‘fascicular pattern’ or ‘appearance’, while others refer to ‘fascicle depiction’^14–16^. This discrepancy arises from the lack of detailed studies with robust methodologies and histological verification to support such claims. A key limitation of HRUS in depicting fascicles is the limited understanding of fascicular dynamics, particularly the merging of fascicles, and the proportion of fascicles that HRUS can accurately visualize. Preliminary studies focusing on the median, ulnar, or sciatic nerve have demonstrated the feasibility of cross-referencing HRUS with other imaging techniques, such as MRM and HCS^6,17,18^. While these microscopic techniques can provide more detailed depictions of nerve fascicles, their requirement for tissue excision limits their applicability in living subjects.

This study sought to provide referential data describing the fascicular anatomy of three upper extremity nerves – median nerve, ulnar nerve, and superficial branch of the radial nerve. To elucidate the HRUS characteristics of these nerves, this study primarily aimed to compare the fascicular anatomy visualized using in situ HRUS with ex vivo imaging modalities: HCS, MRM, and indirectly, OPT. The secondary aim was to evaluate whether referential HCS can be substituted with MRM or OPT for the assessment of nerve morphometric properties.

## Methods

### Ethical approval and cadaveric material

The protocols of this study were approved by the National Medical Ethics Committee of the Republic of Slovenia (Permit No.: 0120–239/2020/3). All experiments were performed in accordance with relevant guidelines and regulations. Seven recently deceased human bodies (5 females and 2 males; mean age, 88.1 ± 7.3 years) with no history of peripheral neuropathy were obtained from the Institute of Anatomy, Faculty of Medicine, University of Ljubljana, Slovenia through a willed cadaver donation program. Written informed consent was obtained from each donor before death. All donors were adults with the capacity to provide consent and were not under guardianship.

None of the subjects had a body mass index over 30 kg/m^2^ or extensive scars and wounds on the upper extremities that could hinder the HRUS protocol. Both upper extremities, including the scapula and clavicle, were harvested from each donor. The specimens were kept in a cold environment. HRUS imaging was performed 30–48 h postmortem to avoid *rigor mortis*.

### Ultrasound imaging with suture positioning

An Arietta 850 ultrasound (US) machine (Fujifilm Visual Sonics, Toronto, Ontario, Canada) with an SML44 linear probe (22–2 MHz) was used for the fascicle depiction and HRUS-guided marking of upper extremity nerves^18^. The median and ulnar nerves were depicted in the mid-upper arm, and the superficial branch of the radial nerve was depicted in the upper third of the forearm. Each nerve was punctured and marked using silk braided sutures (USP 3/0, GS 60 mm, straight cutting, non-absorbable, SMI AG, St. Vith, Belgium) which later served for improving the orientation during the nerve excision process and cross-referencing. After puncturing, the US transducer was put near the suture, perpendicular to the nerve course. The nerves were located at a depth of 1–1.5 cm. HRUS parameters such as gain, focus, and depth were adapted to the individual anatomical situation. The US images of approximately 25-mm-long segments were captured as 15-s-long videos with 26 frames per second, saved as raw files, and exported in Adobe Photoshop 2020 (Adobe Systems, San Jose, California, USA).

### Magnetic resonance microscopy imaging

Immediately following HRUS, the nerves were harvested from the upper extremities. The nerve segment adjacent to the suture was excised, and the surrounding connective tissue was carefully removed. Each sample was trimmed to a 16-mm-long segment, with the suture marking the proximal end. Using plastic inserts, each segment was then placed within a 20-mm-wide glass tube. The nerves were positioned supine within the tube, which had been filled with the perfluorinated liquid Galden HT110 (Solvay, Brussels, Belgium). This liquid prevented sample desiccation during prolonged MRM acquisition and ensured the absence of an MRI signal from the liquid^19^.

MRM acquisition was conducted using an NMR/MRI spectrometer (Tecmag, Houston TX, USA) connected to a 400 MHz (magnetic field: 9.4 T) wide-bore vertical superconducting magnet (Jastec Superconductor Technology, Tokyo, Japan) and to microimaging gradient coils (Micro2.5, Bruker, Ettlingen, Germany) with maxim gradients of 650 mT/m per gradient channel. The images were acquired using a 3-dimensional short tau inversion recovery (STIR) imaging sequence with the following parameters: TR/TE = 2900 ms/6 ms; FOV = 19 mm × 9.5 mm × 16 mm; acquisition matrix (x × y × z) = 512 × 256 × 32; inversion time = 415 ms, slice thickness = 500 μm; no interslice gap; number of slices = 32; and 4 signal averages. The temperature inside the MRM system was set at 20 ± 2 °C and the total acquisition time for set of six nerves from one subject was 27 h.

Immediately after MRM scanning, each sample was rinsed with phosphate-buffered saline and bisected into approximately equal 8-mm-long segments. The proximal segments were immediately frozen in liquid nitrogen and stored at -80 °C until further HCS staining, while the distal segments were prepared for OPT.

### Preparation of histologic cross-sections

The proximal nerve segments containing the sutures were used for HCS preparation. The nerves were sectioned into 4–5 μm-thick slices using a Leica CM 1950 cryostat (Leica Microsystems GmbH, Wetzlar, Germany) and stained with hematoxylin and eosin. Approximately one hundred HCS slices were prepared from each sample and visualized using a Nikon Eclipse 80i microscope (Nikon, Tokyo, Japan). Images of the slices were captured at 40 × magnification with a Microscope camera ODC 841 (Kern & Sohn, Balingen, Germany).

### Optical projection tomography

The distal segments were prepared for OPT by immersion in a graded series of methanol solutions (Chem Lab, Zedelgem, Belgium) with concentrations of 25%, 50%, 75%, and 100%, each for 60 min. Subsequently, the samples were then placed in a mixture of 50% methanol and 50% BABB (one part benzyl alcohol and two parts benzyl benzoate; both from Sigma Aldrich GmbH, Taufkirchen, Germany) for 24 h. Finally, the nerves were cleared in BABB for 8–10 weeks. The entire process was conducted at room temperature (20 ± 2 °C).

After clearing, the distal nerve segments were scanned using a custom-made OPT machine that was developed in cooperation with the Politecnico di Milano, Italy, and installed in the Institute of Physiology, Czech Academy of Sciences in Prague^7,20^. A reusable adhesive (Blu Tack, Bostik, Stafford, UK) was used for mounting the samples to a sample holder, and the quartz cell used for imaging was filled with BABB solution. The autofluorescence of the samples was excited with an LED illuminator at 405/10 nm. The emitted signal was collected by 2 × Plan-Apo Infinity-Corrected ELWD objective (Edmund Optics, Barrington, NJ, USA) through a 447/60 nm emission filter onto an EM CCD camera (Andor, Belfast, UK) with a resolution of 1004 × 1002 pixels. Individual projections of the samples were acquired by rotating the sample 360° around its axis with a step angle of 0.9°, thus yielding 400 images per scan. Tomographic reconstructions were then calculated using a filtered back-projection algorithm in NRecon software 1.6.10.2 (Bruker, Billerica, MA, USA). As the length of the samples exceeded the size of the field of view, the projections of all samples were taken in several successive scans. After the reconstruction, all parts were merged using the program Amira 3D (Thermo Fischer Scientific, Waltham, MA, USA).

### Cross-referencing: histologic cross-sections, high-resolution ultrasound, and magnetic resonance microscopy

HCS were considered as the gold standard to which MRM and HRUS cross-sections were cross-referenced. Three to four HCS with minimal procedural artefacts and an inter-slice distance of at least 0.5 mm, were randomly selected. The final number of cross-referenced images depended on the quality of histologic slices. The HCS were cross-referenced to MRM and HRUS with visual inspection based on the geometrical matching of fascicular patterns and suture position^6^.

Cross-referencing and detailed analysis of the cross-referenced images, including the delineation of structures, were performed using the ImageJ software (National Institutes of Health, Bethesda, Maryland, USA). The software was calibrated independently for each modality, and no post-processing methods were applied. Manual delineation of nerves and fascicles was performed using the software. On HCS, fascicles were identified as oval or round basophilic structures surrounded by a thin layer of pale, more eosinophilic perineurium. The surrounding tissue between the fascicles was identified as interfascicular epineurium. On MRM, fascicles were identified as oval or round hyperintense intraneural structures surrounded by a thin, even more hyperintense layer representing the perineurium. On HRUS, fascicles were defined as oval or round hypoechoic intraneural structures surrounded by interfascicular epineurium. The outer hyperechoic rim surrounding the fascicles was considered to represent the epineurium. During manual delineation of structures on HRUS and MRM, adjacent slices were used to facilitate the identification of fascicles and outer nerve borders.

**Fig. 1:**
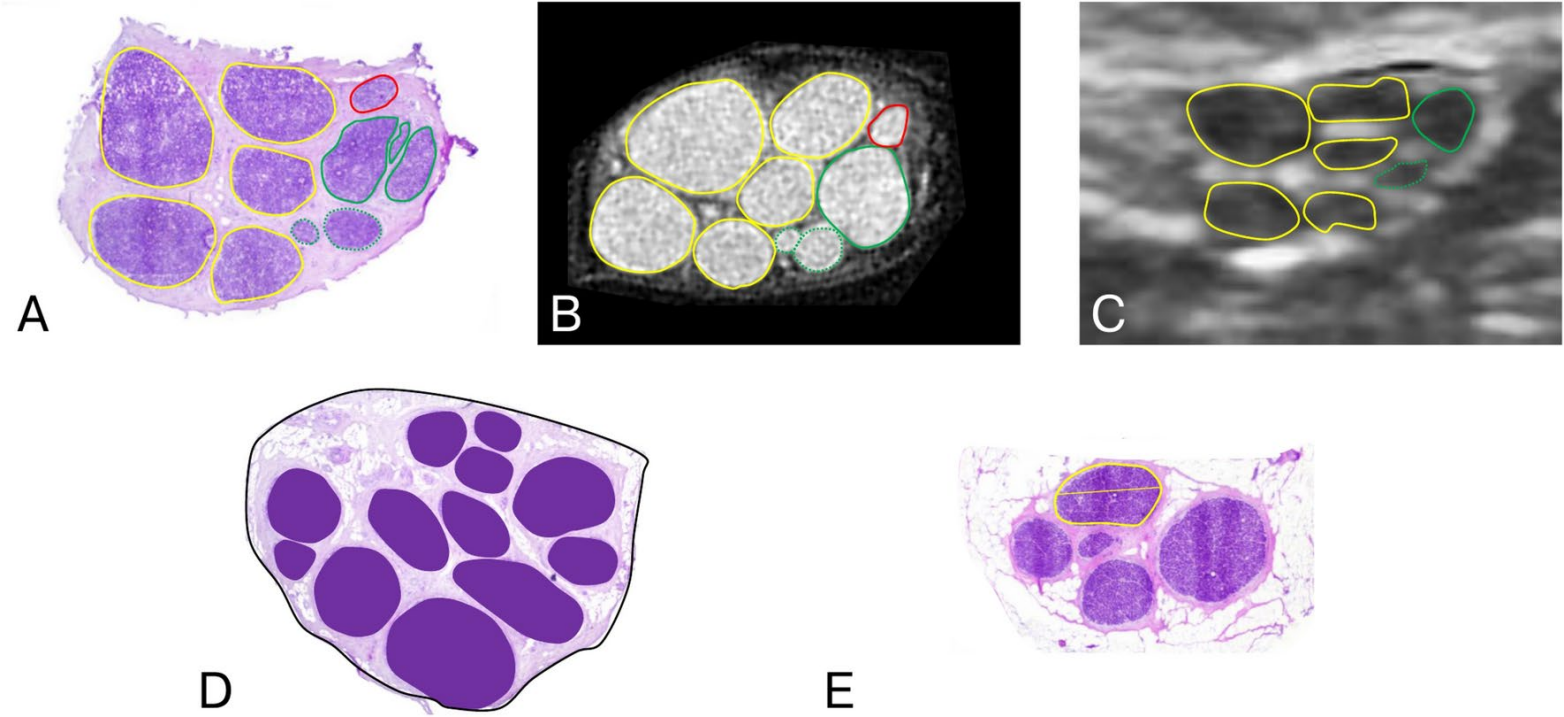
Assessment of fascicle differentiation, fascicular ratio (FR), and roundness. (**A–C**) Fascicle delineation on cross-referenced histological sections (HCS), magnetic resonance microscopy (MRM), and high-resolution ultrasound (HRUS) images. Fascicles delineated in yellow indicate single fascicles (SF), and green highlights fascicular clusters (FC) on MRM and HRUS. One fascicle outlined in red is classified as non-depicted (ND), as it is not visualized on HRUS. (**D**) Calculation of FR, defined as the ratio of the total cross-sectional area (CSA) of all fascicles (purple) to the nerve CSA (black). (**E**) Calculation of fascicle roundness, with CSA and major axis length delineated.

Delineation of the nerve and fascicles on each cross-section allowed for the calculation of the following parameters: fascicular CSA, nerve CSA, roundness of the nerve, roundness of the fascicles, fascicle count, fascicular ratio (FR), and interfascicular epineurium ratio. The fascicle count represented the total number of fascicles recognized on a cross-section. FR was calculated as the total fascicular CSA to nerve CSA ratio (Fig. 1D), while the interfascicular epineurium ratio was calculated as 1 – FR. The roundness (Fig. 1E) of a structure was calculated using the ImageJ plugin (Eq. 1).

1 $$Roundness= \frac{4 \times CSA }{\pi \times {(major\,axis\,lenght)}^{2}}$$

Based on cross-referencing, the fascicles were classified into three groups: single fascicle (SF), fascicular cluster (FC), and no depiction (ND)^6,17^. In the SF group, a fascicle identified as a single structure on HRUS or MRM corresponded to a single fascicle on HCS. Fascicles in the FC group appeared as single structures on HRUS or MRM but corresponded to two or more fascicles on HCS. Conversely, fascicles in the ND group were not identified on HRUS or MRM but were observed on HCS (Fig. 1A-C). To facilitate the comparison between modalities, the accuracy of fascicle depiction was calculated as the ratio between the total number of SF identified on HRUS or MRM and the fascicle count observed on corresponding HCS cross-sections.

### Cross-referencing: magnetic resonance microscopy and optical projection tomography

The cross-referencing of MRM and OPT cross-sections was conducted separately from previous evaluations. Four MRM cross-sections were selected and cross-referenced to OPT based on the fascicle orientation and their size ratio. The OPT cross-sections were selected in a short continuous section of OPT volume where the shape of the nerve was the most circular, regular, and most aligned along the z-axis. The nerve and fascicles were manually delineated as described above. On OPT images, the fascicles were considered oval or round hyperintense structures surrounded by hypointense interfascicular epineurium, while the outer hyperintense rim represented the epineurium. The nerve CSA, fascicle CSA, roundness of nerve, roundness of fascicles, FR, and fascicle count were calculated and compared between the modalities.

### Inter- and intra-observer agreement

Ten cross-referenced HCS, HRUS, and MRM cross-sections and ten cross-referenced MRM and OPT cross-sections were randomly selected, and the manual delineation of the fascicles/nerves was performed by the same observer one month after the initial outlining. To evaluate inter-observed agreement, these cross-sections were additionally evaluated by a second independent evaluator.

### Statistical analysis

Data is presented as mean ± standard deviation (SD) for normally distributed data or as median with interquartile range [IQR] for non-normally distributed data; and as proportions or (ranges). The Shapiro–Wilk test was used to assess the normality of data distributions. For the comparison between the imaging techniques (HCS vs. MRM vs. HRUS), a two-way analysis of variance (ANOVA) for repeated measures was employed on data with normal distribution, followed by Tukey’s *post-hoc* test. For data not following normal distribution, Friedman test was used. Correlations between the accurately depicted fascicles with other analyzed parameters were calculated using linear regression with Bonferroni correction. The simple logistic regression model was employed on HRUS-measured fascicular CSAs for the calculation of cluster probability. The comparison between MRM vs. OPT was performed using repeated measures t-test. The statistical analysis and graphing were performed using GraphPad Prism 10 (GraphPad Software Inc., San Diego, USA). To assess intra- and inter-observer repeatability, the interclass correlation coefficient (ICC) was calculated using SPSS (SPSS Inc., Chicago, Illinois, USA) and interpreted according to the guidelines^21,22^. Inter-observer and intra-observer repeatability for fascicle count, FR, and nerve CSA were assessed separately. Differences were considered statistically significant at *p* < 0.05.

## Results

**Fig. 2:**
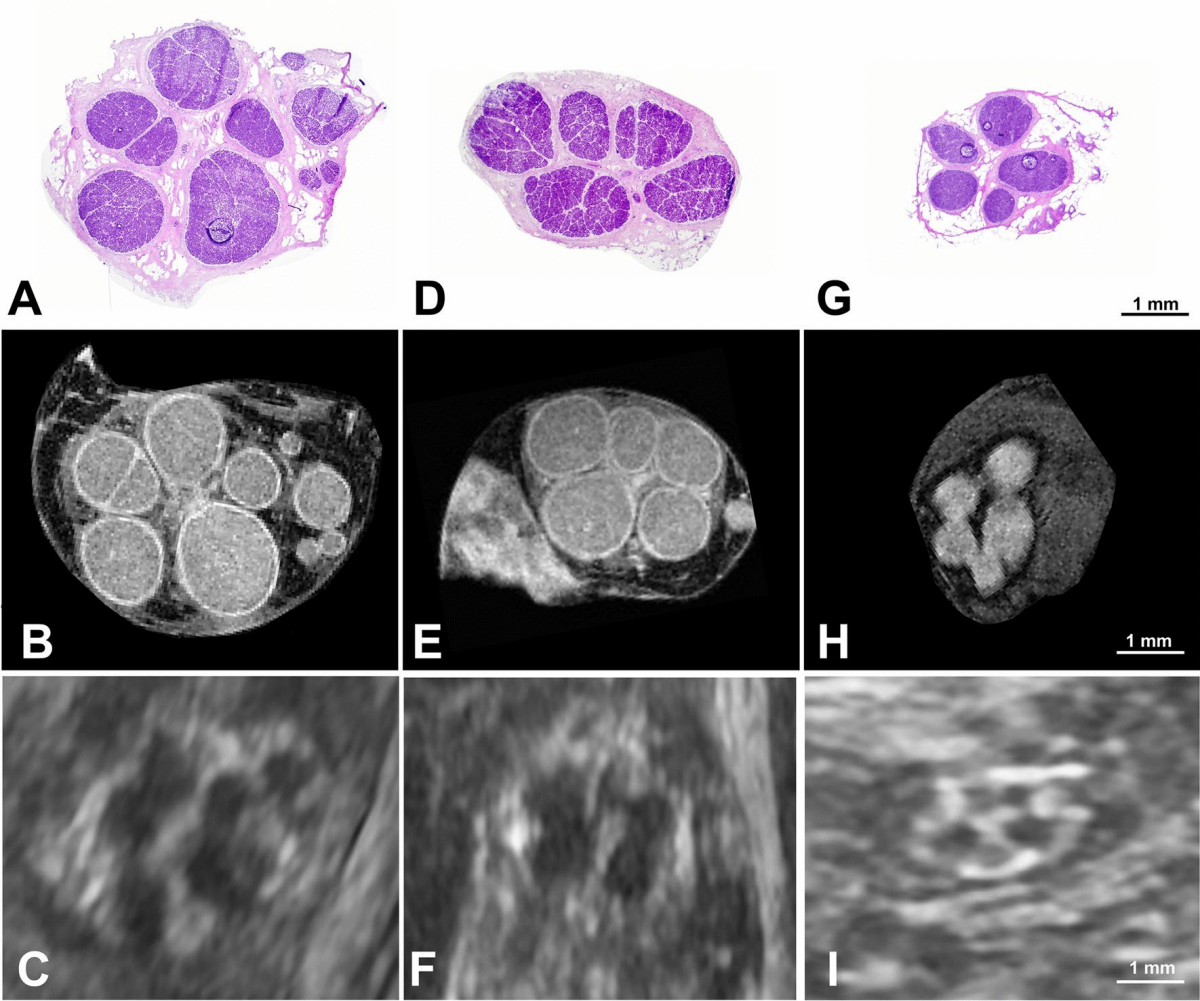
Cross-sectional comparison between imaging modalities. Cross-referencing of (**A–C**) median, (**D–F**) ulnar, and (**G–I**) superficial branch of the radial nerve: a comparison of histological cross-sections (HCS) stained with hematoxylin and eosin (first row), magnetic resonance microscopy (MRM) images (second row), and high-resolution ultrasound (HRUS) images (third row). Scale bar = 1 mm applies to all images.

**Fig. 3:**
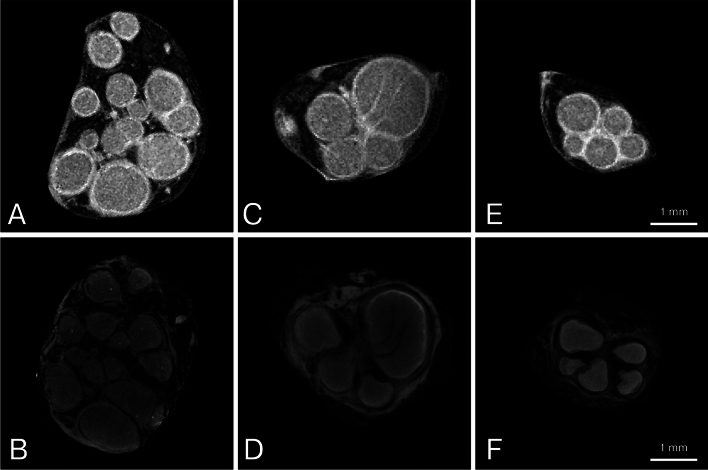
Comparison of magnetic resonance microscopy (MRM, upper row) and optical projection tomography (OPT, bottom row). Corresponding cross-sectional images of (**A, B**) median nerve, (**C, D**) ulnar nerve, and (**E, F**) superficial branch of the radial nerve. Note the altered fascicular patterns on OPT images with hypointense circular halos surrounding individual fascicles. These altered fascicular shapes resulted in reduced roundness and altered distances between adjacent fascicles compared to MRM images. Scale bar = 1 mm applies to all images.

**Fig. 4:**
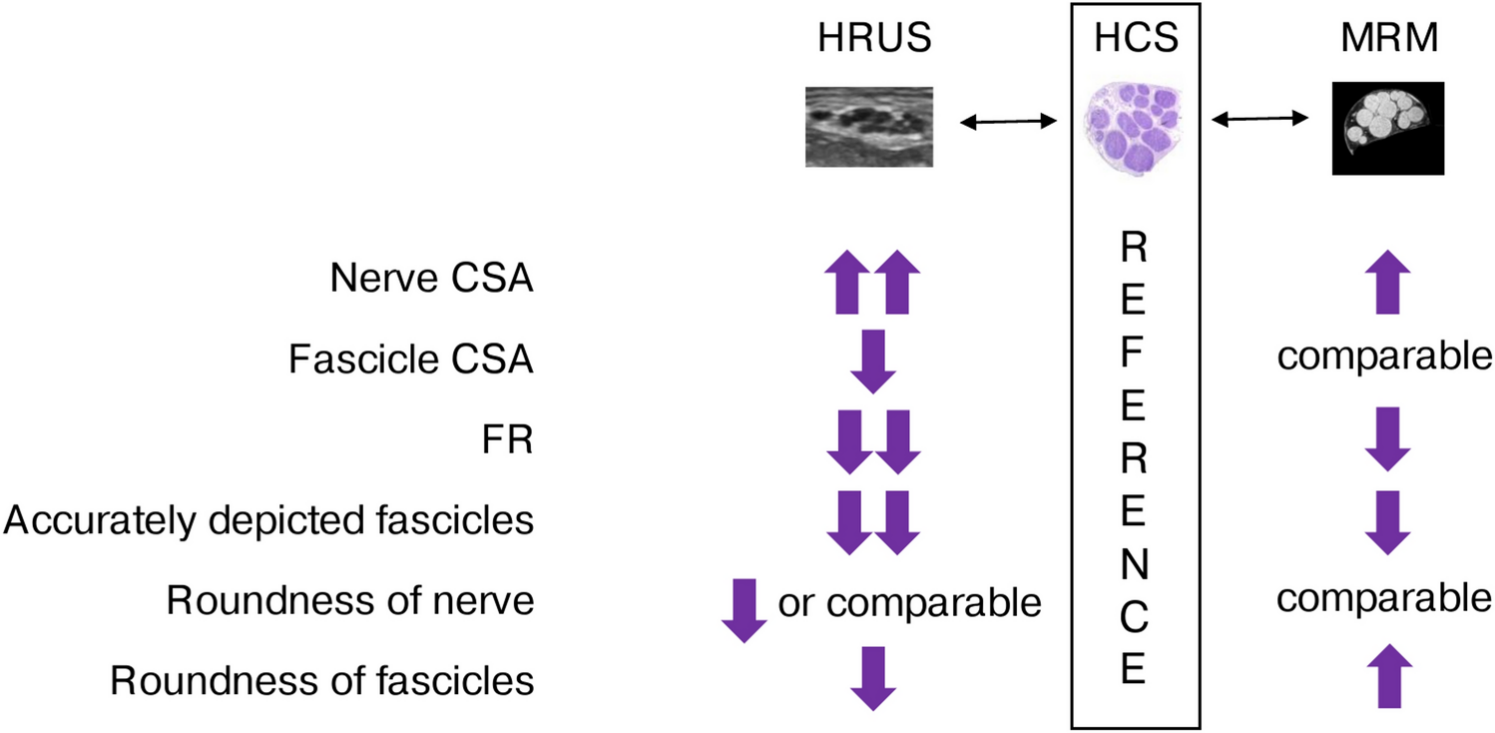
Summary of differences in morphometric parameters between histological cross-sections (HCS), magnetic resonance microscopy (MRM), and high-resolution ultrasound (HRUS). A single upward arrow indicates higher values, and a single downward arrow indicates lower values, compared to HCS. Two arrows indicate the highest or lowest values among the three modalities. CSA, cross-sectional area; FR, fascicular ratio.

A total of 142 cross-sectional images from 41 nerves were cross-referenced between HCS, MRM, and HRUS (Fig. 2), while 164 images were cross-referenced between MRM and OPT (Fig. 3). Results summarizing the comparison between HRUS, HCS, and MRM are presented in Fig. 4.

### Cross-sectional area

The median nerve exhibited the largest nerve and fascicular CSA among the analyzed nerves (Tables 1–3). Repeated measures ANOVA revealed a significant difference in nerve CSA between imaging modalities (*p* < 0.0001), with the highest nerve CSA detected with HRUS for all three nerves. Conversely, the total CSA of all fascicles (per cross-section) was smallest with HRUS and differed significantly from HCS and MRM (*p* < 0.0001). No significant differences in fascicular CSA were observed between HCS and MRM.

**Table 1:** Cross-referenced morphometric properties of histological sections, magnetic resonance microscopy, and high-resolution ultrasound of median nerves.

	HCS	MRM	HRUS
Fascicle count	10.07 ± 2.87	9.19 ± 2.46 (-8.7)	7.04 ± 2.26 (-30.1)
Single fascicles	10.07 ± 2.87	8.61 ± 2.43 (-19.0)	5.40 ± 2.14 (-46.4)
Fascicular clusters	0 [0–0] †	0.58 [0.08–0.67]	1.67 [1.33–2]
Non-depicted fascicles	0 [0–0] †	0.10 [0–0]	0.17 [0–0.46]
Accurately depicted fascicles	1 [1–1] †	0.88 [0.84–0.96]	0.53 [0.48–0.67]
CSA nerve [mm^2^]	9.20 ± 1.73	10.28 ± 1.40 (11.7)	11.08 ± 1.70 (20.4)
CSA fascicles [mm^2^]	5.01 ± 0.89	5.29 ± 0.66 (5.6)	4.33 ± 0.60 (-13.6)
CSA largest fascicle [mm^2^]	1.49 ± 0.85	1.61 ± 0.90 (8.0)	1.60 ± 0.87 (7.4)
Fascicular ratio	0.55 ± 0.09	0.52 ± 0.07 (-5.5)	0.40 ± 0.05 (-27.3)
Interfascicular epineurium	0.45 ± 0.09	0.48 ± 0.07 (6.7)	0.60 ± 0.05 (33.3)
Roundness of nerve	0.62 ± 0.16	0.71 ± 0.11 (14.5)	0.48 ± 0.13 (-22.6)
Roundness of fascicles	0.68 ± 0.08	0.78 ± 0.05 (14.7)	0.57 ± 0.09 (-17.2)

Data calculated for fourteen median nerve samples are presented as mean ± standard deviation (SD) or median with interquartile range [IQR]. Relative differences between modalities are shown in parentheses, with histological data serving as the baseline. CSA – cross-sectional area; HCS – histological cross-section; HRUS – high-resolution ultrasound; MRM – magnetic resonance microscopy; † – considered as a referential value with single fascicles and no cluster fascicles or non-depicted fascicles.

**Table 2:** Cross-referenced morphometric properties of histological sections, magnetic resonance microscopy, and high-resolution ultrasound of ulnar nerves.

	HCS	MRM	HRUS
Fascicle count	7.42 ± 2.42	6.90 ± 2.22 (-7.0)	4.85 ± 1.53 (-34.6)
Single fascicles	7.42 ± 2.42	6.54 ± 2.10 (-11.9)	3.44 ± 1.62 (-53.6)
Fascicular clusters	0 [0–0] †	0.33 [0.67]	1.00 [1.00–2.00]
Non-depicted fascicles	0 [0–0] †	0 [0–0]	0.33 [0–0.67]
Accurately depicted fascicles	1 [1–1] †	0.90 [0.83–0.94]	0.54 [0.45–0.62]
CSA nerve [mm^2^]	5.67 ± 1.09	6.49 ± 1.35 (14.5)	7.48 ± 1.51 (31.9)
CSA fascicles [mm^2^]	3.31 ± 0.59	3.47 ± 0.61 (4.8)	2.94 ± 0.62 (-11.2)
CSA largest fascicle [mm^2^]	1.14 ± 0.56	1.20 ± 0.54 (5.3)	1.24 ± 0.58 (8.8)
Fascicular ratio	0.59 ± 0.07	0.54 ± 0.07 (-8.5)	0.40 ± 0.04 (-32.2)
Interfascicular epineurium	0.41 ± 0.07	0.46 ± 0.04 (12.2)	0.60 ± 0.04 (46.3)
Roundness of nerve	0.66 ± 0.14	0.67 ± 0.06 (1.5)	0.56 ± 0.14 (-15.2)
Roundness of fascicles	0.66 ± 0.08	0.77 ± 0.04 (16.7)	0.58 ± 0.08 (-12.1)

Data calculated for thirteen ulnar nerve samples are presented as mean ± standard deviation (SD) or median with interquartile range [IQR]. Relative differences between modalities are shown in parentheses, with histological data serving as the baseline. CSA – cross-sectional area; HCS – histological cross-section; HRUS – high-resolution ultrasound; MRM – magnetic resonance microscopy; † – considered as a referential value with single fascicles and no cluster fascicles or non-depicted fascicles.

**Table 3:** Cross-referenced morphometric properties of histological sections, magnetic resonance microscopy, and high-resolution ultrasound of radial nerves.

	HCS	MRM	HRUS
Fascicle count	4.45 ± 1.27	4.18 ± 1.37 (-6.1)	3.78 ± 1.16 (-15.1)
Single fascicles	4.45 ± 1.27	4.09 ± 1.43 (-8.1)	3.27 ± 1.24 (-26.5)
Fascicular clusters	0 [0–0] †	0.19 [0–0.33]	0.67 [0.33–0.67]
Non-depicted fascicles	0 [0–0] †	0 [0–0]	0 [0–0.25]
Accurately depicted fascicles	1 [1–1] †	1 [0.84–1]	0.72 [0.67–0.86]
CSA nerve [mm^2^]	1.72 ± 0.34	2.24 ± 0.42 (30.2)	2.31 ± 0.41 (34.3)
CSA fascicles [mm^2^]	0.91 ± 0.21	1.02 ± 0.20 (12.1)	0.70 ± 0.13 (-23.1)
CSA largest fascicle [mm^2^]	0.39 ± 0.19	0.44 ± 0.22 (12.8)	0.33 ± 0.19 (-15.4)
Fascicular ratio	0.53 ± 0.03	0.45 ± 0.06 (-15.1)	0.30 ± 0.04 (-43.4)
Interfascicular epineurium	0.47 ± 0.03	0.55 ± 0.06 (17.1)	0.70 ± 0.04 (49.0)
Roundness of nerve	0.70 ± 0.09	0.64 ± 0.11 (-8.6)	0.56 ± 0.12 (-20.0)
Roundness of fascicles	0.73 ± 0.06	0.79 ± 0.07 (8.2)	0.59 ± 0.08 (-18.9)

Data calculated for fourteen superficial branch of the radial nerve samples are presented as mean ± standard deviation (SD) or median with interquartile range [IQR]. Relative differences between modalities are shown in parentheses, with histological data serving as the baseline. CSA – cross-sectional area; HCS – histological cross-section; HRUS – high-resolution ultrasound; MRM – magnetic resonance microscopy; † – considered as a referential value with single fascicles and no cluster fascicles or non-depicted fascicles.

Differences in nerve and fascicular CSA were also observed when comparing MRM with OPT (*p* ≤ 0.03). OPT exhibited reduced nerve and fascicular CSAs compared to MRM, with mean values 12.2% and 24.2% lower, respectively (Table 4).

**Table 4:** Cross-referenced morphometric properties of magnetic resonance microscopy and optical projection tomography.

	MRM	OPT	Δ (%); *p*
Median nerve			
Fascicle count	8.58 ± 3.27	8.93 ± 3.24	-0.35 (-4.5); ***0.04***
CSA nerve [mm^2^]	9.94 ± 1.63	8.95 ± 1.86	0.99 (10.3); < ***0.01***
CSA fascicles [mm^2^]	5.21 ± 0.64	3.87 ± 0.59	1.34 (25.6); < ***0.0001***
Fascicular ratio	0.53 ± 0.06	0.44 ± 0.08	0.09 (16.5); < ***0.0001***
Roundness of nerve	0.77 ± 0.10	0.73 ± 0.09	0.03 (2.4); *0.43*
Roundness of fascicles	0.78 ± 0.09	0.70 ± 0.05	0.09 (10.0); < ***0.01***
Ulnar nerve			
Fascicle count	5.88 ± 2.26	6.34 ± 2.34	-0.46 (-7.8); < ***0.01***
CSA nerve [mm^2^]	6.44 ± 1.12	5.56 ± 1.35	0.88 (13.6); < ***0.01***
CSA fascicles [mm^2^]	3.71 ± 1.11	2.91 ± 0.80	0.81 (19.2); ***0.03***
Fascicular ratio	0.55 ± 0.06	0.53 ± 0.09	0.02 (3.6); *0.33*
Roundness of nerve	0.72 ± 0.09	0.72 ± 0.12	0 (0); *0.90*
Roundness of fascicles	0.79 ± 0.05	0.72 ± 0.04	0.07 (8.7); < ***0.0001***
Radial nerve			
Fascicle count	5.04 ± 1.53	5.04 ± 1.58	0 (0); > *0.99*
CSA nerve [mm^2^]	2.31 ± 0.41	1.90 ± 0.43	0.41 (17.3); < ***0.001***
CSA fascicles [mm^2^]	1.04 ± 0.20	0.78 ± 0.17	0.26 (24.3); < ***0.0001***
Fascicular ratio	0.45 ± 0.07	0.42 ± 0.08	0.03 (6.2); *0.15*
Roundness of nerve	0.69 ± 0.08	0.73 ± 0.12	-0.04 (-6.0); *0.10*
Roundness of fascicles	0.79 ± 0.07	0.72 ± 0.07	0.07 (8.4) < ***0.001***

Data are presented as mean ± standard deviation. Statistical analysis was performed using a paired t-test for each nerve independently. Δ (%); p – absolute difference (relative difference) between the assessed modalities with magnetic resonance microscopy considered as a baseline value. *p* (value) with bold and italics text indicates a significance; CSA – cross-sectional area; MRM – magnetic resonance microscopy; OPT – optical projection tomography.

### Fascicular ratio

FR was the smallest for cross-sections analyzed with HRUS and highest for HCS (*p* < 0.0001). FR comparison between MRM vs. OPT also revealed significant differences in FR for the median nerve (*p* < 0.0001), while there were no significant disparities when comparing FR of the ulnar or radial nerve (Table 4).

### Fascicle differentiation

The median nerve had the highest fascicle count, while superficial branches of radial nerves had the lowest fascicle count. There were no differences between HRUS and MRM when comparing the number of ND fascicles; however, the differences were significant when comparing the number of FC (*p* ≤ 0.02).

Comparison of the accurately depicted fascicles revealed significant differences between the analyzed modalities (*p* < 0.0001). MRM accurately differentiated 89% (range, 55–100%) of fascicles, while HRUS accurately differentiated 61% (range, 9–100%) compared to the referential HCS (Tables 1–3). Furthermore, a significant difference in accurately depicted fascicles was observed between nerves imaged with HRUS, specifically between the median and radial nerves (*p* = 0.04) and the ulnar and radial nerves (*p* = 0.02), with the radial nerve having the highest proportion of accurately depicted fascicles. There were no linear correlations between the number of accurately depicted fascicles and any other analyzed parameter, except as anticipated, with the number of FC for both MRM (r = -0.91, *p* < 0.0001) and HRUS (r = -0.85, *p* < 0.0001).

**Fig. 5:**
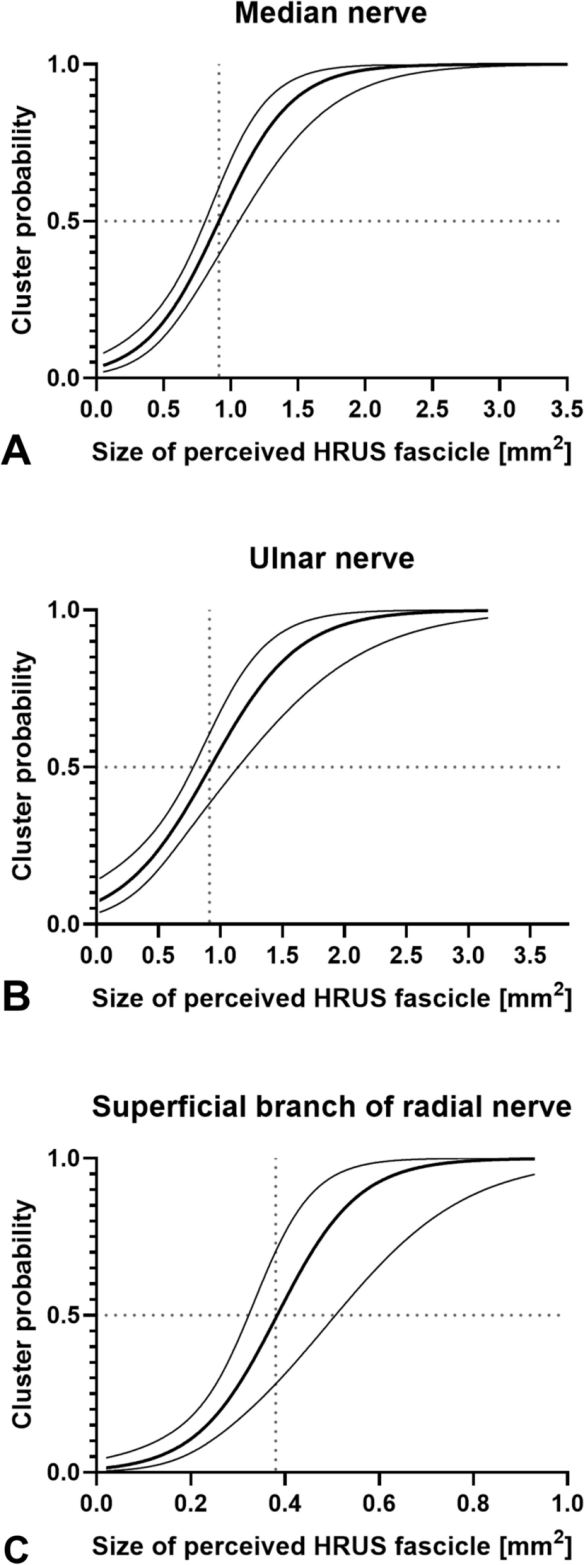
Logistic regression curves of cluster probability on high-resolution ultrasound (HRUS). Logistic regression curves with 95% confidence intervals are shown separately for (**A**) median (R^2^ = 0.36), (**B**) ulnar (R^2^ = 0.23), and (**C**) superficial branch of the radial nerve (R^2^ = 0.35). The intersection of the dotted lines indicates a cluster probability of 0.50. A cluster probability of zero indicates a single fascicle (SF) on the corresponding histological cross-section (HCS), whereas a cluster probability of one indicates that a structure appearing as a single fascicle on HRUS represents two or more fascicles on the HCS. Note the wider 95% confidence interval for the superficial branch of the radial nerve, likely due to the lower number of fascicles assessed in this nerve.

Logistic regression analysis revealed that median and ulnar nerve fascicles with a cross-sectional area larger than 0.91 mm^2^ on HRUS images had a greater than 50% probability of being composed of multiple fascicles on the referential HCS images. For fascicles of the superficial branch of the radial nerve, this threshold was 0.38 mm^2^ (Fig. 5). Fascicles with a cross-sectional area larger than 1.51 mm^2^ (median nerve), 1.70 mm^2^ (ulnar nerve), and 0.57 mm^2^ (radial nerve) on HRUS had a greater than 90% probability of representing a cluster. In most cases, these clusters were composed of two or three fascicles (Table 5). Thirty fascicles with a mean cross-sectional area of 0.03 mm^2^ (range, 0.01–0.08 mm^2^) were not detectable with HRUS, while only eight fascicles, with a mean cross-sectional area of 0.01 mm^2^ (range, 0.01–0.01 mm^2^), were not visualized on MRM.

**Table 5:** Analysis of fascicular clusters in nerve cross-sections visualized with magnetic resonance microscopy and high-resolution ultrasound.

	MRM	HRUS
Fascicular cluster with 2 fascicles; N (%)	34 (83%)	93 (68%)
Fascicular cluster with 3 fascicles; N (%)	6 (15%)	32 (23%)
Fascicular cluster with 4 fascicles; N (%)	1 (2%)	5 (4%)
Fascicular cluster with 5 fascicles; N (%)	0 (0%)	6 (4%)
Fascicular cluster with 6 fascicles; N (%)	0 (0%)	1 (1%)

Data are presented as the absolute number (N) of fascicles with proportion, separately for each modality. HRUS – high-resolution ultrasound; MRM – magnetic resonance microscopy.

OPT proved slightly superior to MRM for visualizing nerve fascicles, with significant differences in fascicle count detected for the median (*p* = 0.04) and ulnar nerves (*p* < 0.01), but not for the radial nerve (Table 4).

### Roundness

Analysis of mean fascicular roundness and nerve roundness revealed significant differences between imaging techniques (*p* < 0.0001 for both). Fascicles on MRM most closely resembled a perfect circle, while those on HRUS exhibited the lowest roundness. Mean fascicular roundness detected with HRUS was, on average, 17% and 26% lower than that observed on HCS and MRM, respectively. Compared to MRM, OPT samples showed significantly altered shapes, reflected in the reduced roundness of the fascicles (*p* < 0.01).

### Intra- and inter-rater agreement

The intra- and inter-rater agreement for manual delineation of nerves and fascicles ranged from good to excellent, depending on the modality (Table 6). The poorest agreement among the assessed modalities was observed for HRUS, particularly for FR.

**Table 6:** Intra- and inter-rater agreement for manually delineating neural and fascicular structures.

	Intra-rater ICC				Inter-rater ICC			
	HCS	MRM	HRUS	OPT	HCS	MRM	HRUS	OPT
Fascicular ratio	0.933	0.910	0.830	0.981	0.910	0.906	0.864	0.951
Nerve CSA	0.999	0.998	0.788	0.988	0.985	0.967	0.879	0.999
No. fascicles	0.997	0.996	0.905	1	0.999	1	0.795	1
Mean	0.976	0.968	0.841	0.989	0.965	0.956	0.846	0.983

CSA – cross-sectional area; HRUS – high-resolution ultrasound; ICC – intraclass correlation coefficient; MRM – magnetic resonance microscopy; OPT – optical projection tomography. ICC models were chosen according to Koo and Li^21^.

## Discussion

In this study, the morphometric characteristics of upper extremity nerves were compared between in situ HRUS and ex vivo imaging modalities: MRM, HCS, and indirectly with OPT. HRUS demonstrated the lowest fascicular CSAs and FRs, but the largest nerve CSAs among the imaging modalities. Compared to the referential HCS, HRUS depicted 51–74% of fascicles. The probability of an HRUS-observed fascicle representing a cluster of fascicles on the referential HCS increased with increasing HRUS-measured fascicular CSA. This probability differed between the larger median and ulnar nerves and the smaller radial nerve. OPT proved marginally superior to MRM in fascicle depiction, while the fascicles visualized on MRM exhibited the greatest roundness.

Significant differences in nerve CSAs were observed between imaging modalities, partially reflecting factors inherent to each modality. Although HCS was considered the gold standard, the potential effects of sample processing on nerve architecture should be acknowledged^23^. Factors such as adipose tissue removal during staining, dehydration of the nerves, or damage to the loose, highly compliant soft tissue during microtome sectioning may contribute to reduced nerve CSAs^24,25^. Therefore, MRM could reflect more accurate, unaltered architecture, as it does not involve tissue processing. However, slightly oblique positioning of the nerve in the MRM probe or sample twisting during acquisition could falsely increase nerve CSA, particularly given the thickness of MRM cross-sections. Conversely, higher nerve CSAs on HRUS were anticipated based on previous studies^26,27^. This could be attributed to the non-perpendicular scanning angles^28^ and, notably, poorly demarcated transitions between the hyperechoic rim of the epineurium and the adjacent perineurium. Additionally, surrounding vessels in situ may be misinterpreted as nerve fascicles^29^, further contributing to increased nerve CSAs.

HRUS-measured FRs were considerably smaller compared to other modalities. This could be attributed to transducer-induced deformation of the nerve or fascicles, or to the HRUS delineation technique, where fascicles were traced within the inner hypoechoic rim. This delineation technique also contributed to the reduced roundness of the fascicles. In addition, falsely larger nerve CSAs due to the inclusion of perineural connective tissue in HRUS images may contribute to a significant reduction in FR. In another pilot study^17^, slightly smaller differences between modalities were observed, suggesting potential influences from different HRUS machines, probes, or delineation techniques. Further investigation is warranted to elucidate these factors, including the effect of semi-automatic tracking analysis^30^. Nonetheless, inter-rater reliability in this study was good to excellent. Notably, the effect of non-perpendicular scanning angles on HRUS or MRM should be less pronounced when comparing FRs, rather than nerve or fascicular CSAs. MRM images effectively delineated the outer epineurium and fascicles, suggesting MRM as a suitable alternative to HCS for evaluating FR, as HCS may be subject to procedural artifacts.

Differences in accurately depicted fascicles on the HRUS between different nerves implicate the possible impact of nerve architecture on fascicular depiction with this modality, presumably reflecting the amount of interfascicular epineurium between the adjacent fascicles^17^. Conversely, the lack of correlation between accurately depicted fascicles on HRUS and fascicle count or other variables suggests that factors unrelated to nerve architecture, such as those related to the ultrasound technique itself, may have a greater impact on fascicle differentiation with HRUS. The use of ultra-high-resolution ultrasound (ultra-HRUS) has shown promise in improving fascicle count accuracy, especially in distal segments of upper and lower extremity nerves where the number of fascicles increases substantially^31–33^. Ultra-HRUS with 48-MHz transducers were even able to differentiate a similar number of fascicles within the superficial branch of radial nerve compared to HCS in our study^34^. Subject-related properties, such as subcutaneous adipose tissue thickness or anatomical position of the nerve, can also hinder fascicle differentiation^6^. The donors in this study were selectively chosen, partly based on body mass index; therefore, the findings regarding HRUS fascicle differentiation should be interpreted cautiously when applied to obese individuals. Adipose tissue, with its signal attenuation and increased acoustic impedance compared to the surrounding skeletal muscle, can reduce image resolution and hinder the visualization of individual nerve fascicles^35^. Therefore, the number of accurately detected SF on HRUS is expected to be lower in obese individuals.

When comparing the fascicle differentiation among the modalities, MRM demonstrated superiority over HRUS. Few fascicles were not depicted on MRM, and the majority of observed clusters were large, partly due to the greater thickness of MRM images. Reducing the image thickness in MRM could potentially enhance its ability to accurately depict more fascicles, although this may require a longer acquisition time or a reduced field of view^36^. With such enhancements, MRM may approach the accuracy of OPT in visualizing fascicles. The larger fascicles observed on HRUS likely represented a cluster of smaller, indistinguishable fascicles, presumably due to the lower resolution and volume-averaging effects inherent to HRUS^26,27,37^. The differences in cluster probabilities between the larger median or ulnar nerves and the smaller superficial radial nerves suggest that cluster formation could be influenced by nerve CSAs.

A further challenge is the lack of generalizability to individuals with peripheral neuropathies, where various pathological processes can cause edema, fibrous tissue proliferation, or even alterations in axonal size and number. These changes lead to alterations in morphometric characteristics such as nerve diameter and CSA, FR, and even echogenicity^38^. In specific neuropathies such as multifocal motor neuropathy, enlargement of individual fascicles can be expected;^39^ therefore, differentiating single fascicles from fascicular clusters is crucial. Cross-referencing HRUS morphometric properties with microscopic techniques in individuals with neuropathies remains an important area for future research. A thorough understanding of fascicle clustering could help establish baseline values for nerve fascicles, but further validation and data on pathological nerves are needed. As obtaining biopsy samples with HCS correlations can be challenging in living subjects, using clinically available 7 T high-field MRM systems could be beneficial for correlating HRUS findings with other diagnostic modalities^40^.

A significant drawback of OPT was the presence of oblique transections, which hindered the accurate assessment of CSAs and roundness, necessitating careful selection of images for analysis. However, the impact of oblique sectioning is minimal and increases gradually for small angles, with deviations of only 1.5% for a 10-degree angle and 6% for a 20-degree angle, proportional to the inverse cosine or cosine of the oblique angle. Roundness not only reflected the fascicle shape induced by sample processing^41^, but also the irregularly contoured interfascicular branches or newly formed fascicles. This makes OPT unsuitable as a reference standard for evaluating this parameter. Despite the shorter scanning time, thinner cross-sections with a theoretical resolution of around 15 μm^25^, and ability to precisely reconstruct interfascicular branches^7^, the issue of oblique sectioning remains a limitation. A significant drawback of OPT compared to MRM is the extended clearing period required for sample preparation. For some samples, this period can take several weeks^42^ Therefore, the marginally better resolution of OPT compared to MRM may not outweigh its time-consuming nature, making it less suitable for evaluating nerve morphometric properties. The reconstructions of fascicles can easily be rendered with OPT, MRM, or tomographic HRUS^43,44^; however, external factors such as preparation artifacts or probe-induced pressure should be considered, potentially altering CSAs or fascicular shapes^45^. 3D fascicular reconstructions can also be rendered from histological slides; however, such methods are time-consuming, require extensive post-processing, and their reliability is questionable, particularly due to sample deformation^46,47^.

This study has some limitations. First, the evaluation of HRUS and MRM images was partly subjective, especially in categorizing ND and FC fascicles. However, this subjectivity did not substantially affect the number of accurately depicted fascicles. Additionally, some of these small fascicles could also be classified as interfascicular connections. Second, a direct comparison between HRUS and OPT was not performed. This omission was due to the difficulty in establishing geometrical alignment between corresponding images, compounded by the absence of sutures or other markers for cross-referencing in the distal nerve portion. Third, nerves from the left and right upper extremities were considered independent samples. Although different extremities exhibited different fascicular patterns, HRUS images were not entirely independent and were partly influenced by cadaver-specific factors, such as subcutaneous adipose tissue content. To mitigate this influence, HRUS scans were intentionally not performed completely symmetrically on the left and right limbs. Finally, the smooth and indistinct transition between the external epineurium and perineural connective tissue hindered the delineation of nerve CSA on HRUS and OPT. Consequently, these measurements were less reliable and subject to inter-observer variability.

In conclusion, 22-MHz HRUS differentiated more than half of the fascicles in the median, ulnar, and radial nerves in non-obese individuals without peripheral neuropathy. Larger fascicular CSA measured on HRUS was associated with a higher probability of representing a cluster of multiple fascicles. Although HCS was considered the gold standard for cross-referencing, the potential impact of sample processing on CSAs and roundness should be acknowledged. MRM and OPT could serve as alternative reference modalities to HCS, depending on the parameters being evaluated.

## Data Availability

Raw data are available upon reasonable request from the corresponding author.
